# Using the Diagnostic Odds Ratio to Select Patterns to Build an Interpretable Pattern-Based Classifier in a Clinical Domain: Multivariate Sequential Pattern Mining Study

**DOI:** 10.2196/32319

**Published:** 2022-08-10

**Authors:** Isidoro J Casanova, Manuel Campos, Jose M Juarez, Antonio Gomariz, Marta Lorente-Ros, Jose A Lorente

**Affiliations:** 1 AIKE Research Team (INTICO) Computer Science Faculty University of Murcia Murcia Spain; 2 Murcian Bio-Health Institute (IMIB-Arrixaca) Murcia Spain; 3 CIBERFES Fragilidad y Envejecimiento Saludable Madrid Spain; 4 Amazon Research Madrid Spain; 5 Department of Medicine Mount Sinai St Luke's-Roosevelt Hospital Icahn School of Medicine at Mount Sinai New York, NY United States; 6 Intensive Care Unit University Hospital of Getafe Getafe Spain; 7 School of Medicine European University of Madrid Madrid Spain; 8 CIBER de Enfermedades Respiratorias Instituto de Salud Carlos III Madrid Spain; 9 Department of Bioengineering Universidad Carlos III Madrid Spain

**Keywords:** sequential patterns, survival classification, diagnostic odds ratio, burn units

## Abstract

**Background:**

It is important to exploit all available data on patients in settings such as intensive care burn units (ICBUs), where several variables are recorded over time. It is possible to take advantage of the multivariate patterns that model the evolution of patients to predict their survival. However, pattern discovery algorithms generate a large number of patterns, of which only some are relevant for classification.

**Objective:**

We propose to use the diagnostic odds ratio (DOR) to select multivariate sequential patterns used in the classification in a clinical domain, rather than employing frequency properties.

**Methods:**

We used data obtained from the ICBU at the University Hospital of Getafe, where 6 temporal variables for 465 patients were registered every day during 5 days, and to model the evolution of these clinical variables, we used multivariate sequential patterns by applying 2 different discretization methods for the continuous attributes. We compared 4 ways in which to employ the DOR for pattern selection: (1) we used it as a threshold to select patterns with a minimum DOR; (2) we selected patterns whose differential DORs are higher than a threshold with regard to their extensions; (3) we selected patterns whose DOR CIs do not overlap; and (4) we proposed the combination of threshold and nonoverlapping CIs to select the most discriminative patterns. As a baseline, we compared our proposals with Jumping Emerging Patterns, one of the most frequently used techniques for pattern selection that utilizes frequency properties.

**Results:**

We have compared the number and length of the patterns eventually selected, classification performance, and pattern and model interpretability. We show that discretization has a great impact on the accuracy of the classification model, but that a trade-off must be found between classification accuracy and the physicians’ capacity to interpret the patterns obtained. We have also identified that the experiments combining threshold and nonoverlapping CIs (Option 4) obtained the fewest number of patterns but also with the smallest size, thus implying the loss of an acceptable accuracy with regard to clinician interpretation. The best classification model according to the trade-off is a JRIP classifier with only 5 patterns (20 items) that was built using unsupervised correlation preserving discretization and differential DOR in a beam search for the best pattern. It achieves a specificity of 56.32% and an area under the receiver operating characteristic curve of 0.767.

**Conclusions:**

A method for the classification of patients’ survival can benefit from the use of sequential patterns, as these patterns consider knowledge about the temporal evolution of the variables in the case of ICBU. We have proved that the DOR can be used in several ways, and that it is a suitable measure to select discriminative and interpretable quality patterns.

## Introduction

### Overview

Advances in the collection and storage of data have led to the emergence of complex temporal data sets, in which the data instances are traces of complex behavior characterized by time series of multiple variables.

In the clinical domain, patients who have incurred severe burns are treated in intensive care burn units (ICBUs). The first 5 days are fundamental: there is a resuscitation phase during the first 2 days and a stabilization phase during the following 3 days, and the patient’s evolution (incomings, diuresis, fluid balance, pH, bicarbonate, base excess) is registered over this period. These variables are not considered in scores for mortality prediction and may play a relevant role in improving the current knowledge of the problem.

Designing algorithms that are capable of learning patterns and classification models from such data is one of the most challenging topics in data mining research [1]. One approach to deal with this problem is discovering patterns that are used as predictors in classification algorithms [2].

The number of patterns initially generated is usually very large, but only a few of these patterns are likely to be of interest to the domain expert that analyzes the data. There are several reasons for this: many of the patterns are either irrelevant or obvious, many patterns do not provide new knowledge regarding the domain, and many of them are similar or are included in others. Measures of the level of interest are, therefore, required to reduce the number of patterns, thus increasing the utility, usefulness, and relevance of the patterns discovered [3]. Some of these interestingness measures are based on the statistical significance of discriminative patterns.

In addition to traditional multidimensional analysis and data mining tasks, one interesting task is that of discovering notable changes and comparative differences. This leads to gradient mining and discriminant analysis [4].

Discriminative pattern mining is one of the most important techniques in data mining. This challenging task comprises a group of pattern mining techniques designed to discover a set of significant patterns that occur with disproportionate frequencies in different class-labeled data sets [5]. Research on discriminative patterns evolves rapidly under several nonuniform definitions, such as contrast sets, emerging patterns, or subgroups. However, these definitions are actually equivalent because their target patterns can be used interchangeably with the same ability to capture the differences between distinct classes [5].

The exploration of discriminative patterns generally includes 2 aspects: frequency and statistical significance. On the one hand, the frequency of a pattern can be assessed by its support, which is defined as the percentage of transactions (in our case, patients) that this pattern contains. A pattern is frequent if its support value is higher than a given threshold.

On the other hand, the statistical significance of discriminative patterns can be measured by using various statistic tests. A pattern is deemed significant if its significance value generated from a certain statistical measure could meet certain user-defined conditions, for example, no less (or more) than a given threshold. Any statistical measure that is capable of quantifying the differences between classes, such as the odds ratio, information gain, or chi-square, is generally applicable, and the choice of this measure will not typically affect the overall performance of the discriminative pattern discovery algorithms [5].

Many specific quantitative indicators of diagnostic test performance have been introduced into the clinical domain, such as sensitivity and specificity, positive and negative predictive values, chance-corrected measures of agreement, likelihood ratios or area under the receiver operating characteristic curve (AUC), among others. But there is a single indicator of diagnostic performance, denominated as the diagnostic odds ratio (DOR), which is closely linked to existing indicators, facilitates the formal meta-analysis of studies on diagnostic test performance, and is derived from logistic models [6].

We propose and compare 4 approaches in which the DOR is used as a statistical measure to select a reduced number of patterns, and we put forward the use of these patterns as predictors in a classification model. The calculation of the DOR for a pattern enables us to use a terminology that is closer to the language of clinicians, in which a pattern is considered to be a risk factor or to have a protection factor.

The first approach consists of using the DOR as a minimum threshold with which to select patterns. In the second approach, we calculate the difference in the DOR of a sequential pattern with respect to its extensions, and we establish a threshold for this difference to reduce the number of patterns selected. One advantage of this approach is that it can be used as an early pruning within the pattern discovery algorithm. In the third place, we calculate a CI for the DOR, and use this CI to prune patterns that are not statistically different from their extension patterns. Finally, we combine the second and third approaches to select patterns with different properties.

We have verified that these propositions provide acceptable results by building a model for the classification of patients’ survival using their daily evolution in an ICBU, employing multivariate sequential patterns. We have additionally compared the 4 approaches with the selection of patterns founded on classical frequency-based measures such as Jumping Emerging Patterns (JEPs).

### Background

#### Sequential Pattern Mining

A sequence database is based on ordered elements or events, recorded with or without a concrete notion of time. There are many applications involving sequence data, such as economic and sales forecasting, speech or audio signals, web click streams, or biological sequences. The mining of frequently occurring ordered events or subsequences as patterns was first introduced by Agrawal and Srikant [7] and has become a significant challenge in data mining.

The purpose of sequential pattern mining is to discover interesting subsequences in a sequence database, that is, sequential relationships between items that are of interest to the user. Various measures can be used to estimate how interesting a subsequence is. In the original sequential pattern mining problem, the support measure is used. The support (or absolute support) of a sequence *s* in a sequence database is defined as the number of sequences that contain *s*, and is denoted by *sup*(*s*).

Sequential pattern mining is the task of finding all the frequent subsequences in a sequence database. A sequence *s* is said to be a frequent sequence or a sequential pattern if and only if *sup*(*s*)≥*minsup*, for a threshold *minsup* established by the user. The assumption is that frequent subsequences are of interest to the user.

With regard to the algorithms employed to mine sequential patterns, there are 3 pioneer proposals: the GSP algorithm with the a priori strategy [8]; the SPADE algorithm, an a priori–based sequential pattern mining algorithm that uses vertical data format [9]; and PrefixSpan with the pattern growth strategy [10]. A number of algorithms based on these 3 proposals have focused on improving their efficiency using different search strategies or data structures.

The researchers refer the reader to [11] for more general information about sequential pattern mining.

#### Pattern and Sequence-Based Classification

Classification rule mining attempts to discover a small set of rules in the database to form an accurate classifier.

Initial approaches that combined pattern mining and classification models employed a strict stepwise approach, in which a set of patterns was computed once and those patterns were subsequently used in models. However, a large number of methods were later proposed, whose aim was to integrate pattern mining, feature selection, and model construction [12].

Some of these are Classification Based on Predictive Association Rules (CPAR), Classification Based on Multiple Association Rules (CMAR) [12], Multi-class, Multi-label Associative Classification (MMAC), and Classification Based on Associations (CBA). Many experimental studies have shown that these integrated classification methods have a high potential approach that builds more predictive and accurate classification systems than traditional classification methods such as decision trees [13].

The classification of sequence patterns is one of the most popular methodologies whose power has been demonstrated by multiple studies [14], and which has a broad range of real-world applications. In medical informatics, the classification of electrocardiogram time series (the time series of heart rates) shows whether the data originates from a healthy person or from a patient with heart disease [15], whereas in financial systems, transaction sequence data in a bank are classified for the purpose of fighting money laundering [16].

The sequence classification methods can be divided into 3 large categories [14]:

- The first category is that of feature-based classification, during which a sequence is transformed into a feature vector, after which conventional classification methods are applied. Feature selection plays an important role in this kind of methods.
- The second category is sequence distance–based classification. The distance function that measures the similarity between sequences determines the quality of the classification in a significant manner.
- The third category is model-based classification, such as using the hidden Markov model and other statistical models to classify sequences.

Conventional classification methods, such as neural networks or decision trees, are designed to classify feature vectors. One way to solve the problem of sequence classification is to transform a sequence into a vector of features by means of feature selections. Sequences can be classified by employing conventional classification methods, such as support vector machine and decision trees.

Several researchers have worked toward building sequence classifiers based on frequent sequential patterns. Lesh et al [17] proposed an algorithm for sequence classification using frequent patterns as features in the classifier. In their algorithm, subsequences are extracted and transformed into sets of features. After feature extraction, general classification algorithms such as support vector machine, naïve Bayes, or neural network can be used for classification. Their algorithm is the first attempt to combine classification and sequential pattern mining.

Tseng and Lee [18] proposed a Classify-By-Sequence (CBS) algorithm to combine sequential pattern mining and classification. Two algorithms, namely, “CBS Class” and “CBS All,” were proposed in their paper. In “CBS Class,” the database is divided into a number of subdatabases according to the class label of each instance. Sequential pattern mining is then implemented on each subdatabase. In “CBS All,” a conventional sequential pattern mining algorithm is applied on the whole data set. Weighted scoring is used in both algorithms.

With regard to the ICBU, few studies have dealt with the problem of survival prediction using machine learning or intelligent data analysis [19].

#### Interestingness Measures for Sequence Classification

In the original sequential pattern mining problem, the main measure used is support. The assumption is that frequent subsequences are of interest to the user.

A first important limitation of the traditional sequential pattern mining problem is that a huge number of patterns may be generated by the algorithms, depending on how the *minsup* threshold is set and on the characteristics of the database [11]. Finding too many patterns could hamper the effectiveness in some cases to which other measures could be better suited.

Many other rule interestingness measures are already used in data mining, machine learning, and statistics. Geng and Hamilton [20] have gathered together 9 different criteria that specify the interestingness of a pattern. These 9 criteria are conciseness, generality, reliability, peculiarity, diversity, novelty, surprisingness, utility, and actionability. These authors additionally classify these criteria into 3 main categories: objective, subjective, and semantics-based measures. Objective measures are those that depend only on raw data. Subjective measures are those that consider the users’ background knowledge in addition to data, and finally semantic-based measures are a special type of subjective measures that take into account the explanation and the semantic of a pattern which are, like subjective measures, domain specific.

In this paper we focus on the probability-base objective measures used in the clinical domain. Some examples of objective rule interestingness measures that are often used in epidemiology as a statistical metric are presented in Table 1.

**Table 1:** Usual clinical objective rule interestingness measures for rules in the form of A→c.

Measure	Formula
Support	*P*(*Ac*)
Confidence	*P*(*c|A*)
Coverage	*P*(*A*)
Prevalence	*P*(*B*)
Specificity	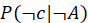
Accuracy	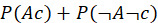
Diagnostic odds ratio	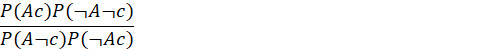
Relative risk	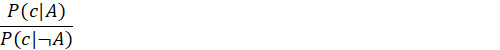

Relative risk and the DOR are statistical metrics that are often used in epidemiological studies. They are consistent: a larger odds ratio leads to a larger relative risk, and vice versa. Under the rare disease assumption, the DOR approximates the relative risk [21]. The DOR is usually used in case-control studies.

Li et al [21,22] used an epidemiological metric, relative risk, to measure pattern interestingness, and concluded that it is an optimal measure to find high-risk patterns. The proposed method was more efficient in covering the search space and produced a smaller number of rules. However, the number of rules in the output could still be too large for an easy interpretation. The authors applied the method to a real-world medical and pharmaceutical–linked data set and it revealed some patterns that are potentially useful in clinical practice.

Most of the conventional frequent pattern–based classification algorithms follow 2 steps [23]. The first step consists of mining a complete set of sequential patterns given a minimum support, while the second consists of selecting a number of discriminative patterns with which to build a classifier. In most cases, mining a complete set of sequential patterns in a large data set is extremely time-consuming, and the huge number of patterns discovered signifies that pattern selection and classifier building are also very time-consuming.

In fact, the most important consideration in sequence classification is not that of finding the complete rule set, but rather that of discovering the most discriminative patterns. In this respect, more attention has recently been paid to discriminative frequent pattern discovery for effective classification.

Heierman et al [24] presented a new data mining technique based on the Minimum Description Length principle, which discovers interesting features in a time-ordered sequence. Petitjean et al [25] introduced a method with which to exactly and efficiently identify the *k* most interesting patterns in a sequential database for which the difference between its observed and expected frequency is maximum: a measure denominated as leverage. Other authors focused on measures for the selection of patterns, such as the relative risk or a coverage measure [26].

In the clinical domain, univariate frequent episodes of Sequential Organ Failure Assessment (SOFA) subscores during the first days after admission were identified in Toma et al [27]. The authors then selected a reduced number of patterns using Akaike’s information criterion to build a logistic regression model to predict the survivability of patients with multiorgan failure. Later, Toma et al [28] showed that the use of univariate patterns as predictors is at least as effective as clinical scores.

After mining JEPs, Ghosh [29] used coupled hidden Markov learning models to build robust sequential patterns–based classifiers. This made it possible to predict hypotension risk, an acute hypotensive episode, or even of a septic shock, with the measurements of the mean arterial pressure, the heart rate, and the respiratory rate.

### Survival Prediction in Intensive Care Burn Units

ICBUs are specialized units in which the main pathologies treated are inhalation injuries and severe burns. Early mortality prediction after admission is essential before an aggressive or conservative therapy can be recommended. Severity scores are simple but useful tools for physicians when evaluating the state of the patient [30]. Scoring systems aim to use the most predictive premorbid and injury factors to yield an expected likelihood of death for a given patient. Baux and Prognostic Burn Index scores provide a mortality rate by summing age and the percentage of total burn surface area, while the Abbreviated Burns Severity Index also considers gender and the presence of inhalation injuries.

The evolution of other parameters during the resuscitation phase (first 2 days) and during the stabilization phase (3 following days) may, however, also be important. The initial evaluation and resuscitation of patients with large burns that require inpatient care can be guided only loosely by formulas and rules. The inherent inaccuracy of formulas requires the continuous reevaluation and adjustment of infusions based on resuscitation targets. Incomings, diuresis, fluid balance, acid-base balance (pH, bicarbonate, base excess), and others help define objectives and assess the evolution and treatment response.

In the ICBU, a patient’s evolution is registered but not considered in scores for mortality prediction. In a previous paper [31], we used emerging patterns with a knowledge-based temporal abstraction and then built classifiers of the survival of the patients with a high sensitivity and specificity. The results of the classification tests showed that our approach is comparable to the burn severity scores used currently by physicians.

## Methods

### Sequential Patterns

Let *I* = {*i*_1_, *i*_2_, ..., *i_k_*} be a set of items. An itemset is a non-empty subset of *I*. A sequence 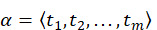 is an ordered list of itemsets 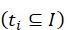 (also called elements or events). Items within an element are unordered and would be listed alphabetically. An item can occur in an element of a sequence once at the most, but can occur multiple times in different elements of a sequence.

The number of instances of items in a sequence is denominated as the length of the sequence. A sequence with a length *k* is called a k-sequence. For example, 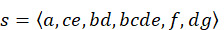 is a sequence that consists of 7 distinct items {a, b, c, d, e, f, g} and 6 itemsets. The length of the sequence is 12 items.

Each itemset in a sequence represents the set of events that occur at the same time (same timestamp). A different itemset appears at a different time.

Sequence 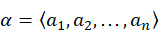 is a subsequence of sequence 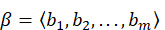 (or *β* is a super-sequence of the sequence α), denoted as 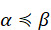, if there exist integers *i*_1_ < *i*_2_ < ^…^ < *i_n_* such that 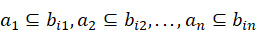. For example, 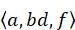 is a subsequence of *s*.

The temporal representation of the patterns is principally carried out using time point representation or time interval representation.

In the time interval representation, there are different ways in which to relate intervals to each other, of which the best known is Allen’s interval algebra [32] or the Time Series Knowledge Representation. In Allen’s interval algebra, there are 13 relations that configure a very expressive language, thus making the pattern representation and the tasks related to temporal reasoning much more complicated.

Time point–based data are a special case of the time interval–based data, in which both the beginning and the end points occur at the same time (for each interval) and the relations between these points become simpler (before, equals or co-occurs, and after), usually denoted as (<, =, >). Furthermore, because the “after” operator (>) is the inverse of the “before” relation (<), if we always consider a relation from the point that occurs first, it is not necessary to use the “after” relation. For instance, if we have A>B, we will instead say B<A.

It is, therefore, possible to define patterns or sequences with only these 2 relations (<, =). Two patterns *a* and *b* are exactly equal if their points are exactly the same and they have exactly the same relations in the same positions, that is, 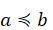 and 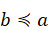.

We have used the FaSPIP algorithm [33] to discover multivariate sequential patterns. FaSPIP is based on the equivalence classes strategy and is able to mine both points and intervals. Moreover, FaSPIP uses a new candidate generation algorithm based on boundary points and efficient methods to avoid the generation of useless candidates and to check their frequency.

In candidate generation, FaSPIP distinguishes between 2 operations to extend a sequence with an item, thus creating a new sequence: Sequence extensions (S-extensions), when the frequent points take place after, and Itemset extensions (I-extensions), when the points take place at the same time as the last item in the pattern. For instance, given the sequence 
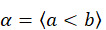
 and a point 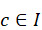, the sequence 
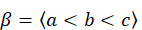
 is an S-extension and 
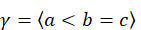
 is an I-extension.

### Emerging Patterns

The classical approach employed for pattern selection is based on the frequency of the patterns. Emerging patterns (EPs) or contrast sets are a type of knowledge pattern that describes significant changes (differences or trends) between 2 classes of data [34]. EPs are sets of item conjunctions of attribute values whose frequency changes significantly from one data set to another. The problem of mining EPs can be expressed as follows: given 2 classes of data and a growth rate threshold, find all patterns (itemsets) whose growth rates—the ratio of their frequency between the 2 classes—are larger than the threshold [3].

Like other rules or patterns composed of conjunctive combinations of elements, EPs can be easily understood and used directly by clinicians.

Furthermore, the concept of JEPs [35] has been proposed to describe those discriminating features that occur only in the positive training instances but do not occur in the negative class at all. The most frequently appearing JEPs have been used to build accurate classifiers [36,37].

### Diagnostic Odds Ratio and CI

Clinicians must rely on the correct interpretation of diagnostic data in a variety of clinical environments. A 2×2 table is an essential tool to present the data regarding epidemiological studies for diagnostic test evaluation (Table 2). The terms commonly used with diagnostic tests are sensitivity, specificity, and accuracy, which statistically measure the performance of the test. *Sensitivity* indicates how well the test predicts one category and *specificity* measures how well the test predicts the other category, while *accuracy* is expected to measure how well the test predicts both categories.

Sensitivity = TP/(TP+FN)

Specificity = TN/(TN+FP)

Other multiple tests with which to improve diagnostic decision making in different clinical situations have also been suggested. For example, Glas et al [6] proposed the use of the DOR as a single indicator of diagnostic performance.

**Table 2:** 2×2 Contingency table.

Test	Reference test	
	Target disorder	No target disorder
Positive	TP^a^	FP^b^
Negative	FN^c^	TN^d^

^a^TP: true positive.

^b^FP: false positive.

^c^FN: false negative.

^d^TN: true negative.

The DOR is used to measure the discriminative power of a diagnostic test: the ratio of the odds of a positive test result among the diseased to the odds of a positive test result among the nondiseased. The DOR is not prevalence dependent, and may be easier to understand, as it is a familiar epidemiological measure. It can be expressed in terms of sensitivity and specificity.

DOR = (TP/FN)/(FP/TN) = [sensitivity / (1–sensitivity)] / [(1–specificity) / specificity]

The value of a DOR ranges from 0 to infinity. To calculate the DOR, the potential problems involving division by 0 are solved by adding 0.5 to the selected cells in the diagnostic 2×2 table.

The further the odds ratio is from 1, the more likely it is that those with the disease are exposed when compared with those without the disease (risk factor). A value of 1 means that a test does not discriminate between patients with the disorder and those without it. Values lower than 1 suggest a reduced risk of disease associated with exposure (protection factor).

CIs for range estimates can be conventionally calculated as shown in the next equation:



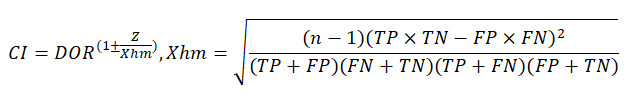



where *Xhm* is the Mantel-Haenszel chi-square and *Z*=1.96 if a confidence of 95% is employed.

Li et al [38] built an algorithm based on the following assumption: if adding an exposure to a rule does not produce a significant change in the DOR, then the rule should not be reported. The DOR between 2 rules is significantly different if their 95% CIs do not overlap.

Several studies based on the nonoverlapping of the DOR have been performed. Toti et al [39] discussed the differences in performance achieved while extracting rules with the different definitions of a nonexposed population, when no pruning criterion is used to filter redundant rules, or when a pruning criterion of redundant rules based on overlapping of 95% CI is added. They confirmed that mining with no pruning criterion produces a high number of redundant rules, thus proving the need for a process with which to eliminate them. Toti et al [40] in another study explained that the traditional interest metrics of support and confidence need to be substituted for metrics that focus on risk variations caused by different exposures. They proposed 2 postprocessing pruning criteria: a rule is pruned if its 95% CI for the DOR crosses the value of 1 or if there is no overlapping of the 95% CI of the rule with all of its parents.

### Case Study

A database contains 480 patient registries, which were recorded between 1992 and 2002. In this database, the temporal attributes that allow the monitoring and evaluation of the response to the treatment of patients are recorded once a day for 5 days. All attributes are continuous variables and represent the value accumulated during 24 hours. The registered variables are (1) total of managed liquids measured in cubic centimeters (cc) represented in the patterns as *INC*; (2) diuresis in cubic decimeters (dc) represented in the patterns as *DIUR*; (3) balance of fluids in cubic decimeters (dc) represented in the patterns as *BAL*; (4) pH; (5) bicarbonate in millimoles/liter (mmol/L) represented in the patterns as *BIC*; and (6) excess base in milliequivalents/liter (mEq/L) represented in the patterns as *BE*. Note that fluid balance is not the difference between revenues and diuresis, but is rather considered to be all the possible eliminations of fluids.

We have removed from the database only those patients who died during the course of the study or those for whom it was not possible to estimate the duration of their hospital stay. After this cleansing, 465 patients remained, of whom 378 patients (81.3%) survived, 324 patients (69.7%) were male, and 201 patients (43.2%) had inhalation injuries. Table 3 provides a summary of the static attributes of the database.

**Table 3:** Attribute summary.

Attribute	Minimum	Maximum	Median	SD
Age (years)	9	95	46.42	20.34
Weight (kg)	25	120	71.05	10.77
Length of stay (days)	3	162	25.02	24.24
Total burn surface area (%)	1	90	31.28	20.16
Deep burn surface area (%)	0	90	17.01	17.41
Simplified Acute Physiology Score	6	58	20.67	9.49

### Experiments

We carried out the experiments by following the 4-step knowledge discovery process described in our previous paper [31]: (1) preprocessing, (2) mining, (3) pattern selection, and (4) classification.

In the first step, the preprocessing was carried out by employing 2 different discretization methods for the continuous attributes. One method was attribute discretization performed by an expert. This method provided the patterns with greater interpretability, because they are expressed in clinical language. The other method is the unsupervised correlation preserving discretization (UCPD), because it provided the best classification in comparison to several automatic discretization algorithms [41].

In the second step, we used the FaSPIP algorithm [33] to discover multivariate sequential patterns. We considered pattern supports ranging from 16% to 6% to find the greatest support that generates the smallest number of patterns with the best classification results. This, therefore, enabled us to obtain interesting patterns, ranging from a small number to thousands of them (Table 4).

The best results were not produced with the lowest supports, which seems to imply that there is no overfitting.

The third step consisted of reducing the number of patterns found to select only those that would be relevant for the classification. If the support used in the previous step is low, the number of frequent patterns increases acutely: the pattern explosion phenomenon is one important disadvantage of using patterns as predictors for classifiers.

We decided to use a baseline experiment to compare it with our proposed methods. We therefore employed the frequency property (because it is frequently used to measure interestingness) to select discriminative patterns. To this end, we selected only JEPs that are not common in the subset of nonsurvivors and survivors, thus enabling us to remove common behavior or a patient’s evolution that is not discriminative.

Finally, the fourth step consisted of building a classification model with the constraint that it had to be interpretable. We wished to obtain a model with a small number of patterns that would be easy for the physician to interpret. In this case, we used a rule learner and a decision tree.

On the one hand, we used Repeated Incremental Pruning to Produce Error Reduction (RIPPER) as a rule learner. With this sequential covering algorithm, rules are learned one at a time, and each time a rule is learned, the tuples covered by the rule are removed. This process is repeated until there are no more training examples or if the quality of a rule obtained is below a user-specified threshold. JRIP (the implementation of RIPPER in WEKA) is one of the best classification algorithms to combine human readability and accuracy [42].

On the other hand, we choose the J48 decision tree implemented by WEKA for the C4.5 algorithm. This employs a greedy technique that is a variant of ID3, which determines the most predictive attribute in each step, and splits a node based on this attribute. Mohamed et al [43] explained that J48 produces high accuracy of classification and simple tree structure. Moreover, Jiménez et al [19] showed that the J48 decision tree algorithm provides the simplest model using the ICBU data set, and thus it is easily interpretable by physicians.

In all cases, we configured the classifiers with the same minimum number of elements in each leaf to 2% and also with the minimal weights of rule instances within a split to 2%. The accuracy, sensitivity, specificity, and AUC were calculated using a 10-fold cross validation.

**Table 4:** Number of interesting patterns selected after mining on the subset of survivors and on the set of nonsurvivors for UCPD^a^ and expert discretization

Discretization and support (%)		Survival + death initial patterns	Baseline JEPs^b^	Experiment 1, DOR^c^		Experiment 2, differential DOR		Experiment 3, nonoverlapping DOR		Experiment 4, differential + nonoverlapping DOR	
				<.08, >16	<.04, >32	All	Best	All	Best	All	Best
**Expert**											
	10	46,041 + 83,015	391	2065	750	2795	2359	858	746	236	198
	8	88,084 + 241,866	4931	14,424	5798	10,655	8781	2195	1856	701	504
	6	224,952 + 492,504	47,113	51,352	41,059	32,406	26,157	4545	3803	1556	1293
**UCPD**											
	16	238,337 + 49,947	2179	14,158	2766	2401	1990	1529	1415	325	272
	14	396,238 + 68,654	7556	33,979	7483	4153	3465	2296	2052	487	411
	12	647,943 + 137,546	22,940	65,564	16,272	9907	8173	6418	5228	1397	1212

^a^UCPD: unsupervised correlation preserving discretization.

^b^JEP: Jumping Emerging Pattern.

^c^DOR: diagnostic odds ratio.

### Ethics Approval

The study was approved by the Ethics Committee of Hospital Universitario de Getafe (38/17, approved on 30/11/2017). This research study was conducted from data obtained for clinical purposes. Informed consent was not required.

## Results

### Overview

The results of the baseline experiment and the results of our 4 different proposals using the DOR are shown below. The number of patterns generated in the subset of survivors and in the set of nonsurvivors with different supports is shown in Table 4. We also studied the length of the patterns produced (Table 5). A short pattern is simpler and more general (it covers more patients). However, a long pattern is more specific (covers fewer patients) and is harder to understand. It is, therefore, more difficult to build a classifier with short patterns.

In the discussion, we explore 3 aspects: classification performance, number and length of patterns selected, and classification interpretability.

**Table 5:** Number (and percentage) of interesting patterns by length (from 2 to 10) for 8% expert discretization and selecting all the patterns when it is possible.

Pattern length	Baseline JEPs^a^ (n=4931)	Experiment 1a, DOR^b^ (<0.08, >16) (n=14,424)	Experiment 1b, DOR (<0.04, >32) (n=5798)	Experiment 2, differential DOR (n=10,655)	Experiment 3, nonoverlapping DOR (n=2195)	Experiment 4, differential + nonoverlapping DOR (n=701)
2	0 (0)	5 (0.0)	0 (0)	289 (2.7)	76 (3.5)	39 (5.6)
3	41 (0.8)	187 (1.3)	49 (0.8)	2063 (19.4)	461 (21.0)	198 (28.2)
4	542 (11.0)	1610 (11.2)	552 (9.5)	3912 (36.7)	857 (39.0)	299 (42.7)
5	1377 (27.9)	4176 (29.0)	1545 (26.6)	3004 (28.2)	612 (27.9)	140 (20.0)
6	1518 (30.8)	4811 (33.4)	1960 (33.8)	1155 (10.8)	175 (8.0)	23 (3.3)
7	987 (20.0)	2698 (18.7)	1190 (20.5)	212 (2)	14 (0.6)	2 (0.3)
8	372 (7.5)	785 (5.4)	407 (7.0)	20 (0.2)	0 (0)	0 (0)
9	84 (1.7)	139 (1.0)	85 (1.5)	0 (0)	0 (0)	0 (0)
10	10 (0.2)	13 (0.1)	10 (0.2)	0 (0)	0 (0)	0 (0)

^a^JEP: Jumping Emerging Pattern.

^b^DOR: diagnostic odds ratio.

### Baseline Experiment: Using JEPs

In the baseline experiment, we searched for discriminative patterns, one of the most important techniques in data mining [44], where the patterns are pruned using only support properties. We selected JEPs, signifying that we maintained patterns found only in the survivors and patterns that occurred exclusively in the nonsurvivors. In a previous paper [31], we verified that this type of emerging patterns produces the best classification results. Furthermore, in this way there is no need to set a threshold that could bring out different results.

Table 6 depicts the results of the experiments carried out using 2 discretization algorithms and by varying the pattern support.

**Table 6:** Results of the baseline experiment with JEPs.^a,b^

Classifier, discretization, and pattern support (%)			Number of patterns	Total length (items)	Average length (items/pattern)	Sensitivity (%)	Specificity (%)	Accuracy (%)	AUC^c^
**J48**									
	**Expert**								
		10	7	33	4.71	100.00	43.68	89.46	0.709
		8	*17*	*84*	*4.94*	*100.00*	*56.32*	*91.83*	*0.782*
		6	16	80	5	100.00	44.83	89.68	0.720
	**UCPD^d^**								
		16	8	29	3.63	100.00	52.87	91.18	0.763
		14	*10*	*37*	*3.7*	*100.00*	*66.67*	*93.76*	*0.853*
		12	12	48	4	100.00	59.77	92.47	0.796
**JRIP**									
	**Expert**								
		10	8	37	4.63	100.00	40.23	88.82	0.704
		8	*15*	*79*	*5.27*	*100.00*	*58.62*	*92.26*	*0.777*
		6	18	87	4.83	100.00	44.83	89.68	0.729
	**UCPD**								
		16	7	34	4.86	100.00	47.13	90.11	0.711
		14	*10*	*35*	*3.5*	*100.00*	*73.56*	*95.05*	*0.866*
		12	12	51	4.25	100.00	62.07	92.90	0.833

^a^JEP: Jumping Emerging Pattern.

^b^Highest specificity is in italics.

^c^AUC: area under the receiver operating characteristic curve.

^d^UCPD: unsupervised correlation preserving discretization.

As will be noted, the JEPs make it possible to achieve a sensitivity of 100%, but the specificity has lower values. This is due to the fact that the data set is imbalanced with a majority of survivors, and the patterns cover only those patients that will survive or those that will die. It is necessary to achieve a higher specificity to predict the nonsurvivors, so the highest specificity is in italics in Table 6 as a baseline best result.

The expert discretization is preferred by clinicians, because it is based principally on reference ranges values. But note that it is possible to improve the results by using an automatic discretization, such as UCPD (see [41]).

When using expert discretization, the highest specificity (58.62%) is obtained using the JRIP classifier with 8% support.

This classifier requires 15 patterns, with a total length of 79 items, with the average length per pattern being 5.27 items. As an example, we show a pattern found in the subset of nonsurvivors. For each variable, the subindex *i* marks the *i* discretization interval where *i*=0 is the lowest interval:

< *BAL*_4_ < *BIC*_1_ < *DIUR*_2_ < *BE*_0_ (10 nonsurvivors, 0 survivors)

There is also an interesting pattern that appears in all the 5 experiments for the subset of nonsurvivors:

< *DIUR*_3_ < *INC*_0_ < *INC*_0_ < *DIUR*_3_ (10 nonsurvivors, 0 survivors)

It would, therefore, be possible to interpret this pattern as “a patient will die if his/her diuresis is very high on one day, and during the next 2 days there is a low income with a very high diuresis the following day.”

### Experiment 1: Using the DOR

In this experiment, we calculated the DOR for each pattern as shown in “Methods” section. In clinical language, a DOR>1 implies that the exposure to the pattern is a risk factor. Conversely, a DOR<1 implies that the pattern is a protection factor and selecting a DOR threshold with a very low value therefore suggests a reduced risk of disease associated with exposure. A value of DOR=1 signifies that the pattern does not discriminate between patients with the disorder and those without it.

The selection of patterns with either a high value or a low value for the DOR will therefore generate discriminative patterns. It is necessary to establish a manual threshold for the value of the DOR to choose the patterns. We have carried out 2 experiments. In the first experiment (1a), we have selected the patterns with a DOR value higher than 16 or lower than 0.08, and in the second experiment (1b), we have selected more exigent values, which were double or half the DOR value, that is, with a DOR value higher than 32 or lower than 0.04. This allowed us to reduce the number of patterns (Table 4) and we obtained a number of patterns in Experiment 1b that were similar to those obtained in the previous experiment. In the more exigent configuration, the length of the selected patterns was almost 6 (Table 5), which was again similar to the baseline experiment.

Tables 7 and 8 show the classification performance of the 2 experiments using expert discretization and UCPD methods with different pattern supports. Expert discretization makes it possible to attain better results than when using JEPs in the previous experiment (Table 6), and worse results than when using UCPD.

**Table 7:** Results of Experiment 1a using the DOR^a^ (<0.08, >16).

Classifier, discretization, and pattern support (%)			Number of patterns	Total length (items)	Average length (items/pattern)	Sensitivity (%)	Specificity (%)	Accuracy (%)	AUC^b^
**J48**									
	**Expert**								
		10	13	67	5.15	90.21	62.07	84.95	0.766
		8	18	89	4.94	88.62	58.62	83.01	0.759
		6	16	80	5	91.80	47.13	83.44	0.702
	**UCPD^c^**								
		16	8	29	3.62	100.00	52.87	91.18	0.763
		14	11	43	3.91	100.00	62.07	92.90	0.787
		12	12	48	4	100.00	59.77	92.47	0.796
**JRIP**									
	**Expert**								
		10	10	46	4.6	91.27	55.17	84.52	0.716
		8	12	58	4.83	93.12	54.02	85.81	0.720
		6	14	67	4.79	94.44	52.87	86.67	0.706
	**UCPD**								
		16	8	33	4.13	100.00	41.38	89.03	0.716
		14	12	47	3.92	100.00	62.07	92.90	0.828
		12	12	46	3.83	100.00	59.77	92.47	0.816

^a^DOR: diagnostic odds ratio.

^b^AUC: area under the receiver operating characteristic curve.

^c^UCPD: unsupervised correlation preserving discretization.

**Table 8:** Results of Experiment 1b using the DOR^a^ (<0.04, >32).

Classifier, discretization, and pattern support (%)			Number of patterns	Total length (items)	Average length (items/pattern)	Sensitivity (%)	Specificity (%)	Accuracy (%)	AUC^b^
**J48**									
	**Expert**								
		10	10	49	4.9	93.65	50.57	85.59	0.710
		8	17	84	4.94	94.18	55.17	86.88	0.767
		6	16	80	5	95.50	37.93	84.73	0.656
	**UCPD^c^**								
		16	8	29	3.62	100.00	52.87	91.18	0.763
		14	11	43	3.91	100.00	62.07	92.90	0.787
		12	12	48	4	100.00	59.77	92.47	0.796
**JRIP**									
	**Expert**								
		10	11	50	4.55	97.09	44.83	87.31	0.704
		8	14	67	4.79	95.50	62.07	89.25	0.801
		6	16	87	5.44	98.15	48.28	88.82	0.715
	**UCPD**								
		16	7	26	3.71	100.00	47.13	90.11	0.727
		14	11	45	4.09	100.00	60.92	92.69	0.792
		12	14	55	3.93	100.00	60.92	92.69	0.822

^a^DOR: diagnostic odds ratio.

^b^AUC: area under the receiver operating characteristic curve.

^c^UCPD: unsupervised correlation preserving discretization.

If we choose expert discretization, with a JRIP classifier and the highest values of the DOR (Table 8), we obtain a higher specificity than with JEPs (62.07%), but a lower sensitivity (95.50%). This can be explained as follows: if we look at one of the 14 patterns used in that classifier, we can find an example of a short pattern with only 3 items:

*BIC*_1_ < *BAL*_4_ < *PH*_1_ (72.30 DOR) (14 nonsurvivors, 1 survivor)

This pattern, with a DOR value of 72.30, classifies a group of patients that will die, although we know that there will be minimal errors (1 patient survives).

We selected the pattern *DIUR*_3_ < *INC*_0_ < *INC*_0_ < *DIUR*_3_ in this experiment because it has a DOR value of 98.05, and it is necessary to recall that all the patients in this pattern will die (10 deaths, 0 survivors). This kind of JEP therefore produces a good specificity, and consequently 100% sensitivity (there are no classification errors).

### Experiment 2: Using the Differential DOR Between a Pattern and Its Extensions

A sequential pattern *p_i_*, of a specific length (*l*), in a point in time (*t*), has a DOR value *DOR*(*p_i_*). In every extension of this pattern (*l*+1), which could be an S-extension (in the next time, *t*+1) or an I-extension (in the same time, *t*), there will be *n* several patterns (*p_i_*_1_, *p_i_*_2_, ..., *p_in_*) that are children of super-pattern *p_i_* with different DOR values, 
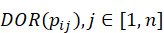
. In this experiment, we choose only the patterns that had a difference in DOR value between the super-pattern and its extensions higher than a threshold *γ*, that is *DOR*(*p_i_*) – *DOR*(*p_ij_*) > *γ*.

For a better interpretation of the DOR, we calculated the risk factor probability *R*(*p_i_*) and the protection factor probability *P*(*p_i_*) as shown in the next equations:

*R*(*p_i_*) = *DOR*(*p_i_*)/[*DOR*(*p_i_*) + 1]

*P*(*p_i_*) = 1 – *R*(*p_i_*)

In our experiment we, therefore, selected the patterns with 2 conditions: (1) when the difference between the risk factor probability *R*(*p_i_*) was greater than 25% or (2) when the difference between the protection factor probability *P*(*p_i_*) was greater than 30%. We chose a lower threshold value for *R*(*p_i_*) because we wished to obtain a higher specificity by having more patterns that were representative of nonsurvivors. In this experiment we obtained patterns with a high quality that produced great changes in the evolution of the patients.

We additionally used 2 alternative strategies to select patterns: it is possible to maintain all the extensions with a difference in the DOR value that is higher than a threshold or to explore the extensions with a beam search, in which case we select only the most promising extension with the highest DOR difference among all extensions. Tables 9 and 10 show the results attained using both strategies.

With regard to the number of patterns selected (Table 4), when we have chosen the best extension, we have only reduced the total number of patterns by less than one-third because the majority of the patterns only have 1 or 2 extensions.

If we study the length of the patterns (Table 5), in this experiment (and in those that follow) the majority of the patterns have a length of around 4, and it is now possible to find more patterns with a shorter length. Note that the distribution of patterns by length has changed. We currently have more general patterns that are shorter. This produces worse classification results when we use expert discretization with a JRIP classifier. It is well known that expert discretization usually performs worse because it is not based on a statistical or information theory that has been specifically designed for classification purposes. This also occurs in almost all of the following experiments.

However, the results obtained with UCPD are similar, and even with the JRIP classification and beam search, we need the lowest number of items and patterns from all the experiments: only 5 patterns with a total length of 20 items are required to attain 56.32% specificity.

**Table 9:** Results of Experiment 2a using the differential DOR^a^ (keeping all pattern extensions).

Classifier, discretization, and pattern support (%)			Number of patterns	Total length (items)	Average length (items/pattern)	Sensitivity (%)	Specificity (%)	Accuracy (%)	AUC^b^
**J48**									
	**Expert**								
		10	28	100	3.57	89.42	49.43	81.94	0.662
		8	21	89	4.24	86.51	62.07	81.94	0.773
		6	18	84	4.67	96.30	44.83	86.67	0.694
	**UCPD^c^**								
		16	21	81	3.86	93.65	49.43	85.38	0.677
		14	15	56	3.73	94.97	56.32	87.74	0.759
		12	12	52	4.33	100.00	58.62	92.26	0.788
**JRIP**									
	**Expert**								
		10	4	13	3.25	90.74	31.03	79.57	0.620
		8	8	25	3.13	86.77	29.89	76.13	0.600
		6	3	7	2.33	89.68	29.89	78.49	0.594
	**UCPD**								
		16	10	37	3.70	92.86	24.14	80.00	0.594
		14	11	41	3.73	94.18	33.33	82.80	0.674
		12	8	26	3.25	96.03	62.07	89.68	0.831

^a^DOR: diagnostic odds ratio.

^b^AUC: area under the receiver operating characteristic curve.

^c^UCPD: unsupervised correlation preserving discretization.

**Table 10:** Results of Experiment 2b using the differential DOR^a^ (using beam search for best pattern extension).

Classifier, discretization, and pattern support (%)			Number of patterns	Total length (items)	Average length (items/pattern)	Sensitivity (%)	Specificity (%)	Accuracy (%)	AUC^b^
**J48**									
	**Expert**								
		10	20	73	3.65	89.15	44.83	80.86	0.642
		8	21	88	4.19	87.57	62.07	82.80	0.783
		6	18	84	4.67	97.35	43.68	87.31	0.710
	**UCPD^c^**								
		16	21	81	3.86	93.65	49.43	85.38	0.675
		14	15	56	3.73	94.71	56.32	87.53	0.760
		12	12	52	4.33	100.00	57.47	92.04	0.764
**JRIP**									
	**Expert**								
		10	18	59	3.28	89.15	27.59	77.63	0.582
		8	5	17	3.4	90.48	21.84	77.63	0.569
		6	8	29	3.62	91.53	31.03	80.22	0.623
	**UCPD**								
		16	9	31	3.44	91.01	28.74	79.35	0.618
		14	19	71	3.74	94.18	34.48	83.01	0.683
		12	5	20	4	97.09	56.32	89.46	0.767

^a^DOR: diagnostic odds ratio.

^b^AUC: area under the receiver operating characteristic curve.

^c^UCPD: unsupervised correlation preserving discretization.

The J48 classification tree used to classify with expert discretization and 8% support, using beam search for the best pattern extension, makes it possible to attain 62.07% specificity, and require 21 patterns, with an average length of 4.19 items per pattern. This average is the lowest value of all the experiments carried out using the J48 classifier with expert discretization. Within these 21 patterns, we can find 2 patterns with only 2 items, which are used to classify the survivors:

*DIUR*_3_ < *BE*_2_ (40.23% PROTECTION) (43 deaths, 150 survivors)

*INC*_2_ = *PH*_3_ (43.58% PROTECTION) (35 deaths, 176 survivors)

The first pattern, *DIUR*_3_ < *BE*_2_, is interesting because if the *PH* is very high the next day and has the extension *DIUR*_3_ < *BE*_2_ < *PH*_4_ (78.85% PROTECTION; 5 deaths, 70 survivors), the patient survival rate increases by 38.62%.

Furthermore, we have discovered a pattern with which to classify the nonsurvivors that can also be found in the J48 tree classifiers of the subsequent experiments, and that was not selected in the classification algorithms used in the previous experiments:

*p_i_*_1_ = *BIC*_1_ < *BIC*_1_ < *PH*_1_ (98.87% RISK; 9 deaths, 0 survivors)

This pattern has a DOR value of DOR(*p_i_*_1_) = 87.12, with a risk probability of *R*(*p_i_*_1_) = 98.87%. It has been selected because its super-pattern *p_i_* = *BIC*_1_ < *BIC*_2_ (44 deaths, 111 survivors) has a DOR value of DOR(*p_i_*_1_) = 2.46, with a risk probability of *R*(*p_i_*) = 71.1%. This signifies that there is an increase in the risk of *R*(*p_i_*_1_) – *R*(*p_i_*_1_) = 27.77%, which is higher than the 25% fixed threshold.

### Experiment 3: Using the Nonoverlapping of the CI of the DOR

In this experiment, we have selected patterns based on the nonoverlapping of 95% CI of the DOR (as stated in [38]). In addition, only patterns whose CI does not include the value 1 have been included in the output (as occurred in [40]). All the patterns are, therefore, either a protector factor or a risk factor, but not both or undetermined.

Table 11 shows the results obtained when we maintain all the pattern extensions, while Table 12 shows the results obtained when only the best pattern extension is chosen using beam search.

We also obtain a reduced number of patterns with respect to the previous experiment (Table 4), and an advantage of this experiment is that this number does not depend on a threshold value.

In general, the classification performance is similar to that of the previous experiments, although with the JRIP classification using expert discretization, we obtain better results when selecting only the best child.

The J48 classification tree used to classify with expert discretization, and 8% support, using beam search for best pattern extension, allows us to obtain 58.62% specificity and a higher sensitivity than the previous experiment: 16 patterns are required.

One of the shortest patterns that we find in the J48 classification tree is:

*PH*_4_ < *PH*_4_ < *BE*_1_ (6 deaths, 1 survivors)

This pattern has a DOR value of 27.93 in the interval (6.71, 116.26). Its super-pattern *PH*_4_ < *PH*_4_ (14 deaths, 109 survivors) has a DOR value of 0.47 in the interval (0.26, 0.87). Note that the CI of these patterns does not overlap.

**Table 11:** Results of Experiment 3a using the nonoverlapping CI of DOR^a^ (keeping all pattern extensions).

Classifier, discretization, and pattern support (%)			Number of patterns	Total length (items)	Average length (items/pattern)	Sensitivity (%)	Specificity (%)	Accuracy (%)	AUC^b^
**J48**									
	**Expert**								
		10	10	41	4.1	93.92	48.28	85.38	0.721
		8	16	77	4.81	94.97	58.62	88.17	0.741
		6	18	90	5	96.56	56.32	89.03	0.768
	**UCPD^c^**								
		16	18	70	3.89	97.35	57.47	89.89	0.794
		14	11	43	3.91	99.74	62.07	92.69	0.803
		12	11	47	4.27	100.00	57.47	92.04	0.786
**JRIP**									
	**Expert**								
		10	11	37	3.36	93.65	41.38	83.87	0.682
		8	13	60	4.62	91.80	33.33	80.86	0.641
		6	7	30	4.29	96.56	42.53	86.45	0.722
	**UCPD**								
		16	6	23	3.83	96.30	41.38	86.02	0.727
		14	9	33	3.67	98.94	56.32	90.97	0.803
		12	14	58	4.14	96.30	60.92	89.68	0.793

^a^DOR: diagnostic odds ratio.

^b^AUC: area under the receiver operating characteristic curve.

^c^UCPD: unsupervised correlation preserving discretization.

**Table 12:** Results of Experiment 3b using the nonoverlapping CI of DOR^a^ (using beam search for best pattern extension).

Classifier, discretization, and pattern support (%)			Number of patterns	Total length (items)	Average length (items/pattern)	Sensitivity (%)	Specificity (%)	Accuracy (%)	AUC^b^
**J48**									
	**Expert**								
		10	10	41	4.1	94.18	51.72	86.24	0.742
		8	16	77	4.81	94.71	58.62	87.96	0.739
		6	18	90	5	96.83	55.17	89.03	0.758
	**UCPD^c^**								
		16	16	68	4.25	96.30	55.17	88.60	0.798
		14	13	51	3.92	100.00	62.07	92.90	0.795
		12	11	45	4.09	100.00	60.92	92.69	0.812
**JRIP**									
	**Expert**								
		10	6	20	3.33	94.44	48.28	85.81	0.735
		8	16	62	3.88	95.24	41.38	85.16	0.700
		6	12	51	4.25	95.77	52.87	87.74	0.747
	**UCPD**								
		16	16	66	4.13	95.50	40.23	85.16	0.695
		14	12	44	3.67	97.88	54.02	89.68	0.747
		12	15	60	4	99.21	55.17	90.97	0.788

^a^DOR: diagnostic odds ratio.

^b^AUC: area under the receiver operating characteristic curve.

^c^UCPD: unsupervised correlation preserving discretization.

### Experiment 4: Using a Differential DOR With the Nonoverlapping of the CI

The last proposal consists of using the previous 2 approaches together (Experiments 2 and 3), signifying that we prune the patterns based on the overlapping of the CI of the DOR, and also based on the difference between the risk (or protection) factor probabilities. In both cases, we maintain the same thresholds.

In this experiment we substantially reduced the number of patterns generated (Table 4). For example, in the case of expert discretization and 8% support (keeping all pattern extensions), we obtained only 701 patterns with this experiment, which is a decrease of 68.06% from nonoverlapping DOR (with 2195 patterns) and a decrease of 85.78% with respect to the baseline experiment (with 4931 patterns).

It is necessary to consider that if the number of patterns is too low, we do not usually achieve a good classification result. But with this experiment, for example, with 8% support, expert discretization, and the J48 classifier, with only 504 patterns, we have obtained a similar result to previous ones, using only 13 patterns in the classifier, with a sensitivity of 96.30% and a specificity of 57.47% in the beam search for the best pattern extension (Table 13). This is the lowest number of patterns required for expert and J48 discretization, with a total length of only 55 items.

The classification performance, as is shown in Tables 13 and 14, is similar to that of the previous experiments.

Let us now analyze the pattern that is selected in this experiment and in all the previous experiments: *DIUR*_3_ < *INC*_0_ < *INC*_0_ < *DIUR*_3_ (10 deaths, 0 survivors). It has a DOR value of 98.05 in the interval (24.21, 397.18), with a risk probability of 98.99%. Its super-pattern *DIUR*_3_ < *INC*_0_ < *INC*_0_ has a DOR value of 2.07 in the interval (1.20, 3.57) with a risk probability of 67.39%, signifying that there is no overlapping in the CI, and that there is an increase in the risk probability of 31.6%.

**Table 13:** Results of Experiment 4b using the differential DOR^a^ and the nonoverlapping CI (using beam search for best pattern extension).

Classifier, discretization, and pattern support (%)			Number of patterns	Total length (items)	Average length (items/pattern)	Sensitivity (%)	Specificity (%)	Accuracy (%)	AUC^b^
**J48**									
	**Expert**								
		10	10	35	3.5	95.50	41.38	85.38	0.694
		8	13	55	4.23	96.30	57.47	89.03	0.770
		6	16	75	4.69	98.41	50.57	89.46	0.739
	**UCPD^c^**								
		16	20	74	3.7	93.92	50.57	85.81	0.758
		14	7	28	4	96.83	58.62	89.68	0.808
		12	12	50	4.17	100.00	59.77	92.47	0.812
**JRIP**									
	**Expert**								
		10	6	21	3.5	92.59	25.29	80.00	0.597
		8	14	43	3.07	91.80	29.89	80.22	0.614
		6	15	57	3.8	92.59	29.89	80.86	0.626
	**UCPD**								
		16	10	37	3.7	96.83	35.63	85.38	0.671
		14	10	36	3.6	98.68	32.18	86.24	0.673
		12	15	59	3.93	98.68	50.57	89.68	0.759

^a^DOR: diagnostic odds ratio.

^b^AUC: area under the receiver operating characteristic curve.

^c^UCPD: unsupervised correlation preserving discretization.

**Table 14:** Results of Experiment 4a using the differential DOR^a^ and the nonoverlapping CI (keeping all pattern extensions).

Classifier, discretization, and pattern support (%)			Number of patterns	Total length (items)	Average length (items/pattern)	Sensitivity (%)	Specificity (%)	Accuracy (%)	AUC^b^
**J48**									
	**Expert**								
		10	13	42	3.23	94.18	44.83	84.95	0.672
		8	13	55	4.23	95.50	55.17	87.96	0.743
		6	17	78	4.59	97.88	47.13	88.39	0.711
	**UCPD^c^**								
		16	20	74	3.7	94.97	50.57	86.67	0.761
		14	7	28	4	98.41	58.62	90.97	0.804
		12	12	50	4.17	100.00	65.52	93.55	0.820
**JRIP**									
	**Expert**								
		10	4	13	3.25	93.12	29.89	81.29	0.622
		8	12	40	3.33	94.44	29.89	82.37	0.625
		6	20	74	3.7	91.80	39.08	81.94	0.668
	**UCPD**								
		16	7	24	3.43	94.44	27.59	81.94	0.632
		14	6	23	3.83	97.35	32.18	85.16	0.653
		12	16	63	3.94	98.68	59.77	91.40	0.795

^a^DOR: diagnostic odds ratio.

^b^AUC: area under the receiver operating characteristic curve.

^c^UCPD: unsupervised correlation preserving discretization.

## Discussion

### Principal Findings

We have proposed different ways of using the DOR as a single indicator of diagnostic performance, by carrying out a classification of the survival of patients in an ICBU by studying their daily evolution using multivariate sequential patterns. We now discuss the factors that we have to consider to have a trade-off mainly between interpretability and classification performance.

In relation to interpretability, a model is more interpretable than another model if its decisions are easier for a human to comprehend than decisions from the other model. In this sense, the presented method shows 3 advantages: (1) the readability and interpretability of the content of the patterns, (2) the reduced length of the patterns, and (3) the small set of significant patterns selected to build the classifier.

Of these 3 advantages, the most direct one for the clinician is that the patterns themselves have an interpretation in the language understood by the clinician, who does not have to spend time looking for a correspondence between what he/she read in the pattern and his/her usual way of working. Moreover, the definition of the patterns provides not only static information about the patient at admission time, as it is usual, but also the evolution of the patient. For example, a pattern like *DIUR*_3_ < *INC*_0_ < *INC*_0_ < *DIUR*_3_ leads the clinician to the clinical factors related to the pattern: high diuresis and very low incomings during 4 different days.

For the second factor, if we study the length of the patterns eventually selected (Table 5), it will be noted that the majority of the patterns in the baseline experiment (using JEPs) and in the first experiment (using DOR) have a length of 6 items, whereas the majority of the patterns in the subsequent experiments have a length of 4 items. We can observe that the distribution of patterns by length has changed, with a larger number of shorter patterns in the last experiments, which are more difficult to use in a classifier, because they are more general. In subsequent Experiments 2-4, we have observed that, on the one hand, the classifier is less accurate. On the other hand, the shorter patterns are easier to understand, more general, and describe the population well, but simultaneously cover survivors and nonsurvivors.

Overall, these shorter patterns produce worse classification results when we use expert discretization with a JRIP classifier. On the one hand, expert discretization generally performs worse, because it is not based on a statistical or information theory that has been specifically designed for classification purposes, and on the other hand, JRIP provides the best performance in terms of the complexity of the tree structure, while J48 produces a high classification accuracy (as the authors explain in [43]). With shorter patterns, however, it is easier to interpret the meaning of the patterns and explain their behavior.

With respect to the third factor, we could say that a model that allows us to achieve a good classification result with a low number of patterns (and consequently of items) is, therefore, preferable. In Table 4 we obtained the smallest number of patterns with Experiment 4 (using a differential DOR and the nonoverlapping of the CI). These patterns are simultaneously restricted by these 2 conditions, and as we have selected a small number of patterns, it might even be interesting to carry out a manual revision and a study of them (although that is out of the scope of this work).

The baseline experiment (using JEPs) and Experiment 3 (nonoverlapping CI of DOR) do not depend on a threshold value and we also obtain a reasonably small number of patterns. Nevertheless the threshold value that has been established in the other experiments (Experiments 1, 2, and 4) leads to changes in the number of patterns eventually selected. We have therefore made 2 variations in Experiment 1 (using DOR), by restricting the minimum DOR value that is necessary to select patterns (Table 8), signifying that we have been able to reduce significantly the appropriate number of patterns selected.

When we work with imbalanced data, as is usual in medical domains, it is necessary to highlight the correct classification of rarely occurring cases when compared with other general cases. It is consequently necessary to check the highest specificity to choose the best classification result, which in our experiments is produced by using UCPD automatic discretization with JEPs as a classical frequency-based discriminative measure. JEPs have usually been used to build accurate classifiers, while UCPD exploits the underlying correlation structure in the data so as to obtain the discrete intervals and ensure that the inherent correlations are preserved.

Moreover, we have generally shown that this automatic discretization performs better classifications than expert discretization. But clinicians prefer to use a reference range discretization for laboratory and physiologic values. This signifies that, for example, they prefer to use the interval (7.35, 7.45) as a normal value for *PH*, as it is usually managed in medicine. The interpretability of the classification results by using expert discretization is, therefore, a prevailing factor in our choice. A summary of the principal results of the experiments using only expert discretization is shown in Table 15.

**Table 15:** Comparison of experimental results with the highest specificity using expert discretization.

Experiment, classifier, and pattern support (%)			Number of patterns	Total length (items)	Average length (items/pattern)	Sensitivity (%)	Specificity (%)	Accuracy (%)	AUC^a^
**JEPs^b^**									
	J48	8	17	84	4.94	100.00	56.32	91.83	0.782
	JRIP	8	15	79	5.27	100.00	58.62	92.26	0.777
**1b: DOR^c^**									
	J48	8	17	84	4.94	94.18	55.17	86.88	0.767
	JRIP	8	14	67	4.79	95.50	62.07	89.25	0.801
**2b: Differential DOR**									
	J48	8	21	88	4.19	87.57	62.07	82.80	0.783
	JRIP	6	8	29	3.62	91.53	31.03	80.22	0.623
**3b: Nonoverlapping DOR**									
	J48	8	16	77	4.81	94.71	58.62	87.96	0.739
	JRIP	6	12	51	4.25	95.77	52.87	87.74	0.747
**4b: Differential + nonoverlapping DOR**									
	J48	8	13	55	4.23	96.30	57.47	89.03	0.770
	JRIP	6	15	57	3.8	92.59	29.89	80.86	0.626

^a^AUC: area under the receiver operating characteristic curve.

^b^JEP: Jumping Emerging Pattern.

^c^DOR: diagnostic odds ratio.

If we therefore consider only expert discretization, the best classification result is achieved in Experiment 1b (using DOR), with a specificity of 62.07% and an AUC value of 0.801 (Table 8). In this experiment we simultaneously obtained patterns found in both the survivors and the nonsurvivors based on only the DOR value of each pattern.

The classification model that is easiest to comprehend and has high specificity requires only 5 patterns (with a total length of 20 items) and is achieved with UCPD and a JRIP classifier in Experiment 2b (differential DOR) using beam search for the best pattern. It obtains a specificity of 56.32% and an AUC value of 0.767 (Table 10).

If we take into consideration only expert discretization, with a J48 classifier we need at least 13 patterns (with a total length of 55 items) to obtain a specificity of 57.47% and an AUC value of 0.770 (Table 13) in Experiment 4b (using a differential and a nonoverlapping DOR).

### Conclusions

In this research, we have developed a model to predict the survival of patients by considering 2 aspects: the relevance of the temporal evolution of the patients as part of the model and an interpretable model for the physicians. We have achieved these aspects by (1) using the multivariate sequential patterns used in classification models that can be easily understood by experts, (2) using a reduced number of patterns, and (3) using a language that is well known by clinicians with regard to both the discretization of values and measures of interest of the patterns.

The main contribution of this work is the proposal and evaluation of 4 ways in which to employ DOR to reduce the number of patterns and to select only the most discriminative ones, because pattern explosion is a principal problem in pattern-based classifiers. We have compared the 4 proposals with a baseline experiment using JEPs. This is, to the best of our knowledge, the first time that some of these approaches have been proposed and compared in scientific literature.

With regard to the number of patterns, the best option is that of using both a differential and a nonoverlapping DOR (as in Experiment 4). As we have increased the restrictions applied, we have significantly reduced the number of patterns, thus attaining more general, simple, and interesting patterns. With expert discretization and 10% support, there are, for example, only 198 patterns (using beam search for best pattern), and, very interestingly, these patterns cover all the patients who did not survive. Despite not being within the scope of this paper, it would be interesting for a clinician to carry out a manual interpretation of these patterns.

This experiment provides the second contribution of this paper, because we have shown that beam search with the DOR could be used in the algorithm to extract sequential patterns for classification rather than using a traditional algorithm for sequential pattern mining.

Despite the efforts made to reduce the amount and the length of patterns in Experiments 2-4, in which we have compared each pattern with its extensions, the classifier built is less accurate. The shorter patterns are easier to understand, more general, and describe the population well, but simultaneously cover survivors and nonsurvivors.

With regard to accuracy, the best classification results are, not surprisingly, produced using JEPs along with UCPD. JEPs have been extensively used to build accurate classifiers and produce better results when we use a discretization based on statistical or information theory that is specifically intended for classification. Nevertheless, we require interpretable patterns that are easy for the clinician to understand, and must therefore use a reference range discretization created by an expert. If we consider only expert discretization, the highest specificity is attained using only the DOR to select the patterns (as in Experiment 1; Table 15).

With regard to interpretability, we can observe that discretization has a great impact on classification performance at the expense of interpretability, because more and longer patterns are required. With UCPD, we require only 5 patterns (with a total length of 20 items) to build a rule set and to obtain 56.32% specificity when we use the differential DOR (see Experiment 2). With expert discretization, we need at least 13 patterns (with a total length of 55 items) to obtain a specificity of 57.47% using both a differential and a nonoverlapping DOR to select the patterns (see Experiment 4).

Our future research will consist of exploring domain-based measures to evaluate clinical patterns or to reduce the number of patterns in postprocessing to an even greater extent. In this respect, we intend to investigate more specific properties, such as closed, maximal, or minimal patterns as a trade-off between improving classification performance and not losing information or representativeness of the population. The researchers additionally intend to explore other measures and search strategies that could be integrated into new algorithms.

## Abbreviations

AUC: area under the receiver operating characteristic curve
CBA: Classification Based on Associations
CBS: Classify-By-Sequence
CMAR: Classification Based on Multiple Association Rules
CPAR: Classification Based on Predictive Association Rules
DOR: diagnostic odds ratio
EP: emerging pattern
FN: false negative
FP: false positive
ICBU: intensive care burn unit
JEP: Jumping Emerging Pattern
MMAC: Multi-class, Multi-label Associative Classification
RIPPER: Repeated Incremental Pruning to Produce Error Reduction
SOFA: Sequential Organ Failure Assessment
TN: true negative
TP: true positive
UCPD: unsupervised correlation preserving discretization

## References

[1] Batal I, Fradkin D, Harrison J, Moerchen F, Hauskrecht M (2012). Mining Recent Temporal Patterns for Event Detection in Multivariate Time Series Data. http://europepmc.org/abstract/MED/25937993.

[2] Bringmann B, Nijssen S, Zimmermann A (2009). Pattern-Based Classification: A Unifying Perspective. http://arxiv.org/abs/1111.6191.

[3] Fan H (2004). Efficient Mining of Interesting Emerging Patterns and Their Effective Use in Classification (PhD thesis). The Department of Computer Science and Software Engineering, University of Melbourne.

[4] Han J, Cheng H, Xin D, Yan X (2007). Frequent pattern mining: current status and future directions. Data Min Knowl Disc.

[5] He Z, Gu F, Zhao C, Liu X, Wu J, Wang J (2017). Conditional discriminative pattern mining: Concepts and algorithms. Information Sciences.

[6] Glas AS, Lijmer JG, Prins MH, Bonsel GJ, Bossuyt PM (2003). The diagnostic odds ratio: a single indicator of test performance. Journal of Clinical Epidemiology.

[7] Agrawal R, Srikant R (1995). Mining sequential patterns. Proceedings of the Eleventh International Conference on Data Engineering.

[8] Srikant R, Agrawal R, Apers P, Bouzeghoub M, Gardarin G (1996). Mining sequential patterns: Generalizations and performance improvements. Advances in Database Technology — EDBT '96.

[9] Zaki MJ (2001). SPADE: an efficient algorithm for mining frequent sequences. Machine Learning.

[10] Mortazavi-Asl B, Pinto H, Dayal U, Jian Pei, Jiawei Han, Jianyong Wang, Qiming Chen, Mei-Chun Hsu (2004). Mining sequential patterns by pattern-growth: the PrefixSpan approach. IEEE Trans. Knowl. Data Eng.

[11] Gan W, Lin JC, Fournier-Viger P, Chao H, Yu PS (2019). A Survey of Parallel Sequential Pattern Mining. ACM Trans. Knowl. Discov. Data.

[12] Li W, Han J, Pei J (2001). CMAR: accurate and efficient classification based on multiple class-association rules. IEEE Xplore.

[13] Nofal M, Bani-Ahmad S (2010). Classification Based on Association-Rule Mining Techniquese a General Survey and Empirical Comparative Evaluation. Ubiquitous Computing and Communication Journal.

[14] Xing Z, Pei J, Keogh E (2010). A brief survey on sequence classification. SIGKDD Explor. Newsl.

[15] Hu B, Chen Y, Keogh E (2013). Time Series Classification under More Realistic Assumptions.

[16] Drezewski R, Dziuban G, Hernik L, Paczek M (2015). Comparison of data mining techniques for Money Laundering Detection System.

[17] Lesh N, Zaki M, Ogihara M (1999). Mining features for sequence classification. Proceedings of the Fifth ACM SIGKDD International Conference on Knowledge Discovery and Data Mining - KDD 99.

[18] Tseng VSM, Lee CH (2005). CBS: A new classification method by using sequential patterns.

[19] Jiménez F, Sanchez G, Juarez JM (2014). Multi-objective evolutionary algorithms for fuzzy classification in survival prediction. Artif Intell Med.

[20] Geng L, Hamilton HJ (2006). Interestingness measures for data mining. ACM Comput. Surv.

[21] Li J, Fu AW-c, He H, Chen J, Jin H, McAullay D, Williams G, Sparks R, Kelman C (2005). Mining risk patterns in medical data. KDD '05: Proceedings of the Eleventh ACM SIGKDD International Conference on Knowledge Discovery in Data Mining.

[22] Li J, Fu AW, Fahey P (2009). Efficient discovery of risk patterns in medical data. Artif Intell Med.

[23] Wu S, Zhao Y, Zhang H, Zhang C, Cao L, Bohlscheid H (2009). Debt Detection in Social Security by Adaptive Sequence Classification. Lecture Notes in Computer Science. Vol 5914 LNAI.

[24] Heierman E, Youngblood M, Cook D (2004). Mining temporal sequences to discover interesting patterns.

[25] Petitjean F, Li T, Tatti N, Webb GI (2016). Skopus: Mining top-k sequential patterns under leverage. Data Min Knowl Disc.

[26] Li I, Huang J, Liao I, Lin J (2013). A sequence classification model based on pattern coverage rate. Lecture Notes in Computer Science, vol 7861. Springer.

[27] Toma T, Abu-Hanna A, Bosman R (2008). Discovery and integration of univariate patterns from daily individual organ-failure scores for intensive care mortality prediction. Artif Intell Med.

[28] Toma T, Bosman R, Siebes A, Peek N, Abu-Hanna A (2010). Learning predictive models that use pattern discovery--a bootstrap evaluative approach applied in organ functioning sequences. J Biomed Inform.

[29] Ghosh S (2017). Multivariate Sequential Contrast Pattern Mining and Prediction Models for Critical Care Clinical Informatics (Thesis). OPUS.

[30] Sheppard N, Hemington-Gorse S, Shelley O, Philp B, Dziewulski P (2011). Prognostic scoring systems in burns: a review. Burns.

[31] Casanova IJ, Campos M, Juarez JM, Fernandez-Fernandez-Arroyo A, Lorente JA (2015). Using Multivariate Sequential Patterns to Improve Survival Prediction in Intensive Care Burn Unit. Lecture Notes in Computer Science, vol 9105.

[32] Allen J (2013). Maintaining Knowledge about Temporal Intervals. Readings in Qualitative Reasoning About Physical Systems.

[33] Gomariz A (2014). Techniques for the Discovery of Temporal Patterns (PhD Thesis). University of Murcia (Spain), University of Antwerp (Belgium).

[34] Dong G, Li J (1999). Efficient mining of emerging patterns. Proceedings of the Fifth ACM SIGKDD International Conference on Knowledge Discovery and Data Mining. KDD '99.

[35] Dong G, Li J, Zhang X (1999). Discovering Jumping Emerging Patterns and Experiments on Real Data sets. http://corescholar.libraries.wright.edu/knoesis/402.

[36] Li J, Dong G, Ramamohanarao K (2001). Making Use of the Most Expressive Jumping Emerging Patterns for Classification. Knowledge and Information Systems.

[37] Dong G, Zhang X, Wong L, Li J (1999). CAEP: Classification by aggregating emerging patterns. Lecture Notes in Computer Science. Vol 1721.

[38] Li J, Liu J, Toivonen H, Satou K, Sun Y, Sun B (2014). Discovering statistically non-redundant subgroups. Knowledge-Based Systems.

[39] Toti G, Vilalta R, Lindner P, Price D (2016). Effect of the Definition of Non-Exposed Population in Risk Pattern Mining.

[40] Toti G, Vilalta R, Lindner P, Lefer B, Macias C, Price D (2016). Analysis of correlation between pediatric asthma exacerbation and exposure to pollutant mixtures with association rule mining. Artif Intell Med.

[41] Casanova IJ, Campos M, Juarez JM, Fernandez-Fernandez-Arroyo A, Lorente JA (2017). Impact of time series discretization on intensive care burn unit survival classification. Prog Artif Intell.

[42] Daud NR, Corne DW (2009). Human readable rule induction in medical data mining. Lecture Notes in Electrical Engineering. Vol 27 LNEE.

[43] Mohamed WNHW, Salleh MNM, Omar AH (2012). A comparative study of Reduced Error Pruning method in decision tree algorithms.

[44] Liu X, Wu J, Gu F, Wang J, He Z (2015). Discriminative pattern mining and its applications in bioinformatics. Brief Bioinform.

